# Soft Tissue Sarcomas Mimicking Benign Inflammatory Processes: A Diagnostic Dilemma

**DOI:** 10.31138/mjr.270823.sts

**Published:** 2023-08-27

**Authors:** Cleofina Furtado, Rania Zeitoun, Jonathan Wilkes, Vaiyapuri Sumathi, George Tony

**Affiliations:** 1 Department of Diagnostic and Interventional Radiology, University Hospitals of North, Midlands NHS Trust, Stoke on Trent, United Kingdom,; 2 Department of Diagnostic and Interventional Radiology, Kasr Al-Ainy Faculty of Medicine, Cairo University, Cairo, Egypt,; 3 Department of Musculoskeletal Pathology, The Royal Orthopaedic Hospital NHS Foundation Trust, Robert Aitken Institute for Clinical Research (Level 3), University of Birmingham, Birmingham, United Kingdom

**Keywords:** synovial chondrosarcoma, malignant transformation, synovial sarcoma, magnetic resonance imaging, computed tomography

## Abstract

**Background:**

Soft tissue sarcomas are rare and often go undetected until a later stage, particularly when they present as intra-articular or tenosynovial lesions mimicking benign synovial pathologies. The failure to distinguish between malignant and benign synovial disease can have a significant impact on patient outcomes and limit alternatives for local control surgery and limb salvage.

**Case Description:**

In this case series, we present two cases of soft tissue sarcomas, one being an intraarticular synovial chondrosarcoma, and the other a pleomorphic spindle cell sarcoma centred along tendon sheaths. Radiologically, the initial clinical presentation of these cases resembled benign synovial pathologies, leading to a delay in diagnosis and treatment.

**Conclusion:**

Our study underscores the importance of maintaining a low threshold of suspicion for surveillance, a multidisciplinary approach, and early histological diagnosis to ensure appropriate timely treatment and a favourable prognosis for patients with soft tissue sarcomas.

## BACKGROUND

Synovial chondrosarcomas (SCH) are rare, slow-growing malignant tumours that arise in synovial tissue.^1^ While they are locally aggressive, they rarely undergo chondrometaplasia.^2^ SCH can arise from primary synovial chondromatosis (PSC), which poses a diagnostic challenge as the two are difficult to distinguish based on radiology alone.^3^ The average time for malignant transformation is reported to be 11.2 years, and the knee is the most commonly affected joint, followed by the hip and ankle.^2^

Synovial sarcoma (SSM) is a high-grade soft tissue sarcoma that frequently occurs around joints, tendons, or bursae, although it can also occur in extra-articular areas.^4^ Mesenchymal spindle cell tumour is a subtype of SSM that exhibits variable epithelial differentiation and a biphasic nature (spindle and epithelial).^5^ SSM is often misdiagnosed as a benign lesion due to its slow growth pattern, benign radiographic appearance, variable size, and capacity to generate pain similar to that caused by common injuries and inflammatory processes.^6^

There is significant overlap between the imaging features of PCS and SCH and between the imaging features of inflammatory tenosynovitis and tendon sheath-based SSM. Misdiagnosing an SSM as a benign lesion can lead to delays in diagnosis and an unfavourable prognosis. However, incorrectly assuming a benign lesion is an SSM can result in patient anxiety and unnecessary intervention.^7^ In this article, we present two cases of soft tissue sarcomas arising from synovial tissue in the knee and wrist with features resembling benign inflammatory synovial processes at initial presentation. Our objective is to highlight the difficulty in distinguishing between benign and malignant pathology in diseases of synovial origin and to emphasise the importance of surveillance, multi-modality imaging, and a multi-disciplinary approach in such cases.

## CASE DESCRIPTION

### Case 1

**Figure 1.**
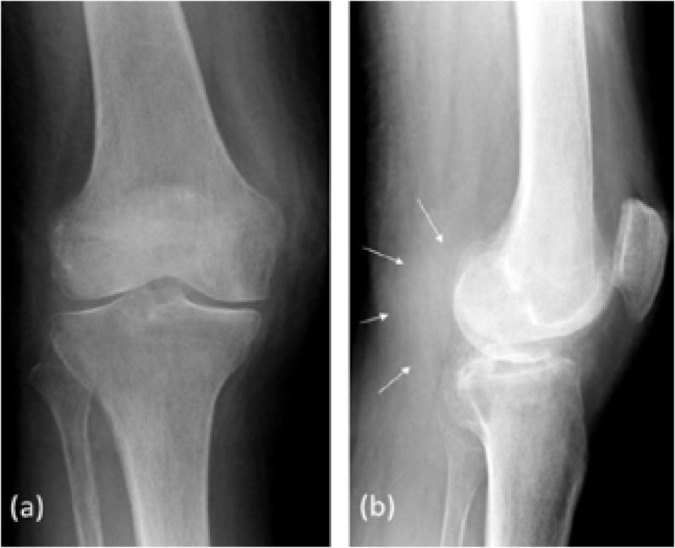
RIGHT knee **(a)** Frontal and **(b)** Lateral X-ray at initial presentation. Osteopenic bony appearances. Small volume suprapatellar joint effusion. Small popliteal fossa soft tissue swelling (arrows). Mild narrowing of joint spaces with no obvious osteophytes. No erosive bony changes.

An 87-year-old female patient presented to the primary care service with a progressively worsening swelling over a period of 18 months. The swelling initially started in the popliteal fossa of her right knee following a right-sided hip replacement, and was initially diagnosed as a Baker cyst. The patient underwent plain X-rays (**Figure 1**), which did not reveal any concerning findings except for minor joint space narrowing and a small popliteal soft tissue swelling. There was no evidence of any peri-articular or intra-articular mineralisation, and no erosions were observed.

Despite a trial of aspiration of the right suprapatellar bursa and anserine bursa in clinic, the patient experienced multiple failed attempts, resulting in dry aspirates. Further investigations through blood tests revealed that the patient’s rheumatoid factor, anti-CCP antibodies, uric acid, and erythrocyte sedimentation rate (ESR) levels were all within normal ranges.

The patient was referred to the bone and soft tumour musculoskeletal clinic due to the progressive nature of the swelling and intractable pain. Upon examination, there was tenderness and swelling in the right knee over the suprapatellar, anserine, and popliteal region, with severe restriction in a fixed flexion position. The swelling was characterised as very tense and firm, but without warmth, redness, or fluid fluctuation. Furthermore, the patient’s knee exhibited severe restriction, with fixed flexion limited to approximately 30 to 40 degrees.

**Figure 2.**
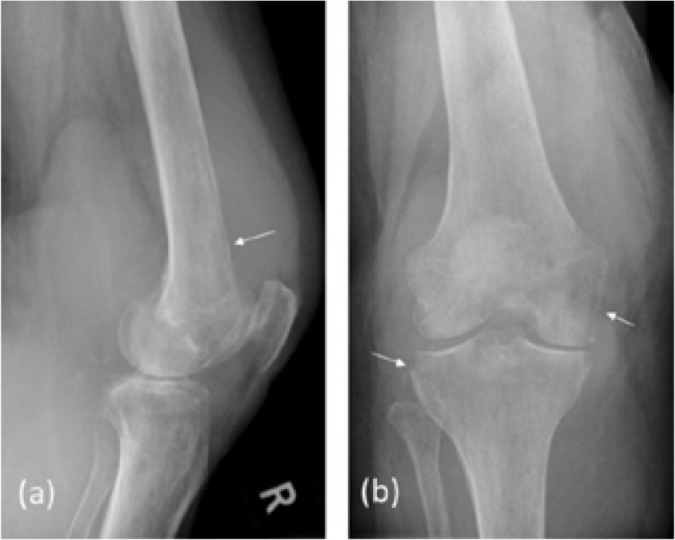
RIGHT Knee Lateral **(a)** and Frontal **(b)** X-ray. Gross supra and infrapatellar soft tissue swelling which had significantly increased in size. Loss of cortical definition to the anterior cortex of the distal femur (arrow in a) and medial femoral condyle suggestive of underlying erosions (arrows in b).

**Figure 3.**
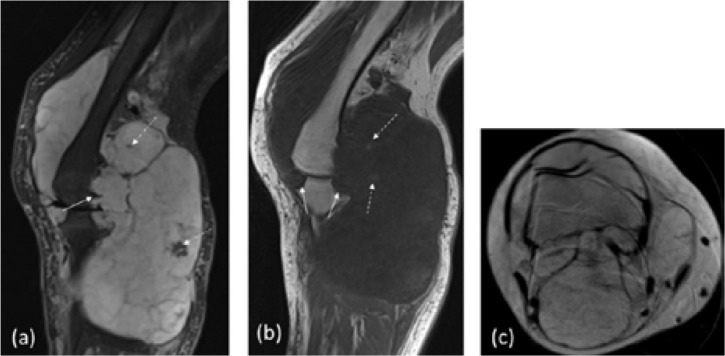
RIGHT knee MRI. Sagittal T2 fat sat **(a)**, sagittal T1 WI **(b)**, axial T2 WI **(c)**. Significant distension of the knee joint capsule extending up into the suprapatellar region and down into the upper one-third of the leg. The material within the joint capsule shows internal signal intensity atypical for fluid, hyperintense on T2 and iso-to-hypointense on T1, with multiple internal heterogeneous septations. Multiple punctate low signal foci seen predominantly along the inferior aspect of the distended joint capsule with a faint T1 hyperintense component suggesting a haemorrhagic component [white dashed arrows in (a) and (b)]. Erosions along the anterior and posterior tibia and the posterior femoral condyle [white solid arrows in (a) and (b)].

**Figure 4.**
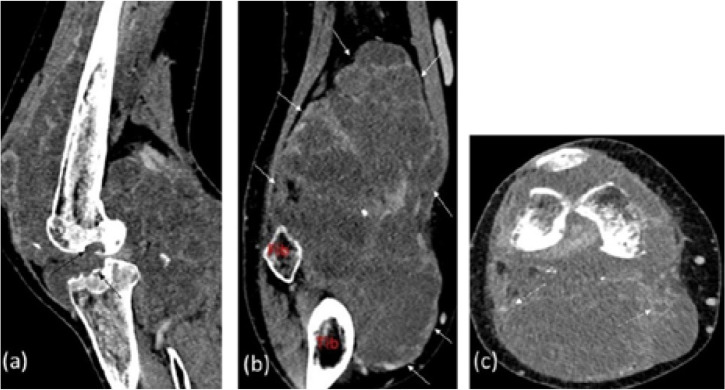
RIGHT Knee CT with post contrast. Sagittal **(a)**, coronal **(b)**, axial **(c)**. Erosive changes and bony destruction (black arrows). There is a large septate lesion with intra and extra-articular components (within white arrows on b) that demonstrates enhancement (dashed white arrows) on the postcontrast imaging predominantly along the septations.

An up-to-date plain X-ray revealed a progressive increase in the gross supra and infrapatellar and periarticular soft tissue swelling, with mild loss of cortical definition in the anterior cortex of the distal femur and medial femoral condyle, and high-density flecks of mineralisation within the peri-articular area of soft tissue swelling (**Figure 2**). A magnetic resonance imaging (MRI) scan was subsequently conducted, which showed marked distension of the knee joint capsule with atypical signal and multiple heterogenous thickened septations, along with multiple bone erosions with well-defined scalloping on the adjacent femur and tibia but strikingly little subarticular bone marrow oedema (**Figure 3**). The T2 gradient echo sequences were not suggestive of pigmented villonodular synovitis (PVNS) or amyloid-based pathology, although there was a small area of haemorrhage seen inferiorly in the posterior component. A marked mass effect was noted with lateral deviation of the femoropopliteal vascular bundle. A multi-slice computed tomography (CT) was also done (**Figure 4**) to confirm the nature of low signal punctate foci which were seen at MRI, which were confirmed to be internal mineralisation.

**Figure 5.**
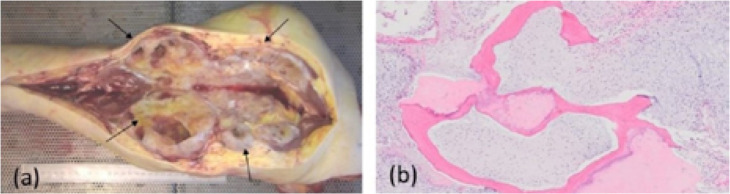
Histopathological images: Macroscopic **(a)**, microscopic **(b)**. RIGHT above knee amputation showing a grey to white cartilaginous tumour (26 x 15 x 14cm) with focal areas of necrosis [between black arrows on (a)]. The tumour is 34cm from the bone resection margin. On Microscopic image (b) grade 3 chondrosarcoma secondary to synovial chondromatosis is seen here infiltrating bone.

In view of the increasing size, dry aspiration, and MRI signal characteristics, which were atypical for pure effusion/synovitis, a synovial soft tissue tumour or malignant process was suspected. She was referred to a tertiary sarcoma centre and planned for a true cut biopsy where histology confirmed grade 3 SCH on a background of PSC (**Figure 5**).

Due to the large tumour size, limited potential benefits from radiotherapy, and lack of meaningful function at the knee joint, the patient underwent a right high above-knee amputation.

### Case 2

**Figure 6.**
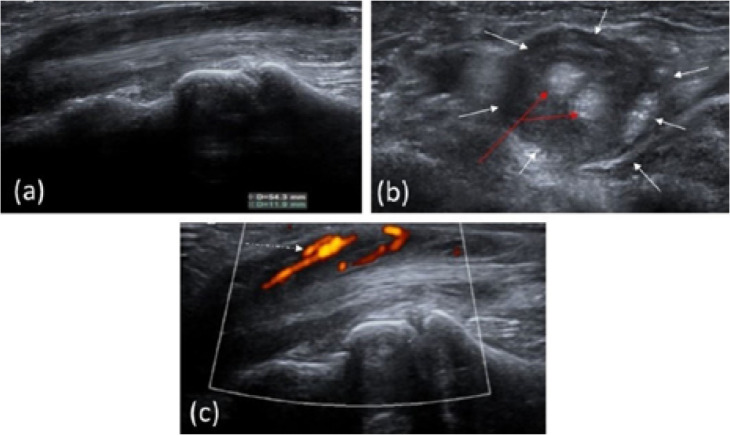
LEFT Wrist Ultrasound. Longitudinal axis **(a)**, Transverse axis **(b)**, power colour doppler **(c)**. Fusiform swelling due to apparent extensive synovial proliferation (within white arrows) is present surrounding flexor digitorum superficialis tendon slips (double red arrows) to the left index and middle fingers. The apparent synovial proliferation extends from just proximal to the wrist to the level of the MCPJs. The colour Doppler images demonstrates hyperaemia (white dashed arrow).

A 68-year-old right-handed female was referred to the Musculoskeletal clinic due to a left wrist injury sustained during her holiday 2 years ago. She had a medical history of a successful trapeziectomy of the left hand and carpal tunnel decompression a couple of years prior to the injury. After the trauma, she experienced pain and spontaneous swelling over the left forearm. An ultrasound scan was conducted, which revealed features of flexor tenosynovitis (**Figure 6**). She received a trial of a steroid injection in her flexor tendon sheath and a course of oral steroids, but neither intervention provided significant relief from her symptoms. Additionally, multiple attempts were made to aspirate the lesion, but these were found to be uncomfortable and yielded dry aspirates.

Before an outpatient routine MRI could be performed, the patient presented with a rapidly increasing swelling in her left forearm, accompanied by increasing pain, crepitus, and functional impairment. Upon examination, the swelling was tense and tender on the volar aspect of the left forearm with overlying erythema of the skin and dilated subcutaneous veins. The patient was experiencing severe pain and had flexion deformity of her fingers.

**Figure 7.**
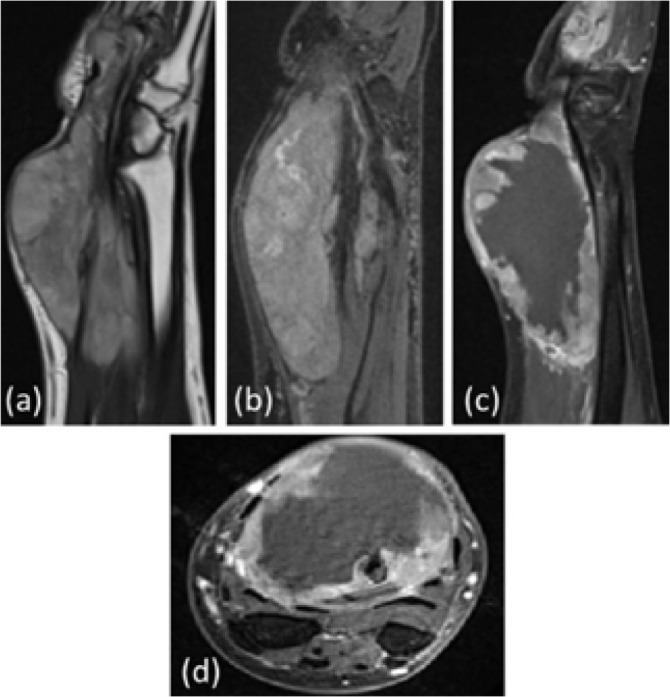
LEFT Wrist MRI. Sagittal T2 WI **(a)**, Coronal T2 fat sat **(b)**, Sagittal T1 fat sat post contrast **(c)**, Axial T1 fat sat with contrast **(d)**. Large volume soft tissue lesion seen within the volar aspect of the left distal forearm measuring 48 x 42 x 98 mm. It demonstrates a heterogeneous signal on the T2 WI sequence with intermediate signal on T1 and demonstrates irregular peripheral enhancement.

**Figure 8.**
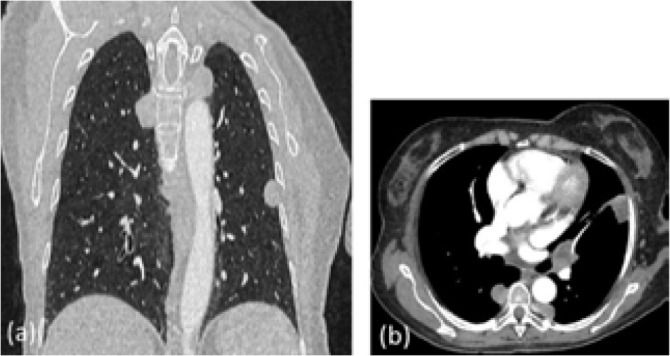
CT chest with contrast. **(A)** Coronal lung window **(B)** Axial soft tissue window. CT thorax with contrast demonstrating multiple rounded soft tissue lesions distributed throughout both lungs in keeping with lung metastases (white arrows).

An urgent MRI was conducted, which revealed a rapidly growing, fungating soft tissue lesion (**Figure 7**). Due to the concerning findings, the patient was transferred to a tertiary sarcoma centre, where a biopsy confirmed the presence of spindle cell/pleomorphic sarcoma. She then underwent a left-sided above-elbow amputation and chemotherapy. A follow-up CT scan of the thorax, abdomen, and pelvis performed after two cycles of chemotherapy revealed progressive diffuse pulmonary metastasis (**Figure 8**).

## DISCUSSION

Synovial-based pathologies pose a diagnostic challenge as they encompass a wide range of both benign and malignant conditions.^8–10^ Malignant pathologies such as SCH and SSM may mimic benign synovial pathologies like synovial cysts, ganglia, fasciitis, bursitis, tenosynovitis, as well as inflammatory processes such as crystal arthropathy, gouty tophi, and rheumatoid nodules.^11–14^ The monophasic and biphasic histo-logic subtypes of SSMs are often misinterpreted as benign pathology due to their slow growth and well-defined appearance.^15^

Patients typically present with a slowly growing mass accompanied by persistent pain despite conservative treatment.^16^

Ultrasound (US) is considered a reliable modality for detecting and diagnosing synovial diseases with good accuracy, particularly in the hands and feet. However, it may not be sufficient to detect suspicious features in deep-seated or intra-articular soft tissue lesions in large joints.^17^ Both inflammatory synovitis and malignant synovial pathology can demonstrate hypoechogenicity and neovascularisation on ultrasound evaluation. X-rays can be useful for demonstrating intra- and peri-articular mineralisation.^18^ Nonetheless, benign pathologies such as synovial chondromatosis and crystal deposition arthropathy, as well as malignant lesions like SCH, can also exhibit this feature.^19^ CT is beneficial for identifying subtle soft tissue calcifications and minor local bone erosions that may not be visible on X-ray.^20^ Conversely, MRI allows for better demonstration of anatomical detail and the extent of such pathology, and can help confirm the solid nature of malignant lesions, especially on post-contrast sequences. It is also superior to both US and X-ray in detecting any features that could raise suspicion of a malignant pathology.^21^

In both presented cases, an initial diagnosis of an inflammatory synovial process was made based on clinical and imaging features. However, due to the clinical course with a progressive increase in size, a prompt action and referral to the sarcoma pathway were taken. A prior case series revealed that 7 out of 17 of the lesions excised either outside of sarcoma service or on the assumption that they were not sinister were later found to be malignant, with 3 of the 7 (43%) having developed metastases due to missed diagnoses.^22^ Following another study in which four patients presented after incomplete resection of a soft tissue sarcoma mistakenly diagnosed as a ganglion cyst, Crosby et al proposed an algorithm for assessing dorsal wrist masses, which includes transillumination and aspiration at the initial clinic visit, MRI, and referral to a specialist centre in atypical lesions.^23^ The pivotal moment at which the suspicion of malignancy was substantially elevated in both of our cases was the orthopaedic specialist evaluation. This emphasises the critical need for early face-to-face surgical review in the suspected sarcoma pathway, ideally before sarcoma multidisciplinary team (MDT) discussion.^19^

In cases of suspected synovial pathology, it is recommended to have a low threshold for follow-up imaging, consider a sinister diagnosis, and refer the patient for further evaluation if the lesion is larger than 5 cm at initial presentation, shows a considerable increase in size when compared to previous imaging, or is associated with persistent or progressive pain.^22–23^ However, the diagnostic challenge arises when these tumours are discovered when they are still small and do not meet the referral criteria to a sarcoma MDT.^22^ The patient’s prior clinical history and the impressions of other professional colleagues may create subconscious bias, which radiologists must be aware of.^23^ Even with advanced diagnostic imaging, an experienced radiologist may miss the initial diagnosis of malignant synovial-based tumours. Therefore, careful collaboration between the clinician and radiologist is essential to re-evaluate the differential diagnosis, maintain a high index of suspicion for malignant pathology, and facilitate an early referral to the sarcoma MDT to improve patient outcomes.^24^

In the management of malignant synovial pathology, surgical resection continues to be the primary treatment modality.^25^ In instances where limb salvage is not feasible due to involvement of the neurovascular bundle, extra-articular excision with large surgical margins or amputation are recommended approaches.^26^ Although adjuvant radiotherapy may be of benefit in high-grade tumours of the limbs that are greater than 5 cm in diameter and deep to the fascia, its effect on survival is unclear. While amputation is an effective means of ensuring local control, it has significant negative consequences on various aspects of a patient’s life.^27^ Hence, early detection and diagnosis of these tumours are crucial to minimise the need for such interventions.^24^

## CONCLUSION

The diagnostic challenge in identifying malignant synovial pathology is compounded by the considerable overlap in clinical and imaging features with benign synovial pathology, such as inflammatory arthropathy. While ultrasound is reliable for synovial diseases in hands and feet, it may miss suspicious features in deep-seated or intra-articular lesions in large joints. Both inflammatory and malignant synovial pathologies can exhibit similar ultrasound findings, and X-rays can demonstrate mineralisation in both benign and malignant conditions. Additional imaging modalities, including CT and MRI, play a crucial role in detecting soft tissue calcifications, bone erosions, and confirming solid malignancies.

It is imperative to consider malignant synovial-based tumours in the differential diagnosis of inflammatory joint disease, particularly when there are atypical clinical features, progressive swelling and pain, and poor response to treatment for inflammation. To achieve an accurate diagnosis, a low index of suspicion, surveillance imaging, a multi-disciplinary approach and early histopathological evaluation are critical. By adopting these measures, effective management can be initiated, and a favourable outcome can be achieved.

## Abbreviations:

CT: Computed tomography
MRI: Magnetic resonance imaging
OP: Outpatient
PSC: Primary synovial chondromatosis
SCH: Synovial chondrosarcoma
SSM: Synovial sarcoma
US: Ultrasound
